# Neighborhood social fragmentation and cerebello-thalamo-cortical connectivity in youth at clinical high-risk for psychosis and healthy comparisons

**DOI:** 10.1016/j.braen.2026.100016

**Published:** 2026-03-10

**Authors:** Benson S. Ku, Ella J. Arrant, Jean Addington, Carrie E. Bearden, Kristin S. Cadenhead, Tyrone D. Cannon, Ricardo E. Carrion, Matcheri S. Keshavan, Daniel H. Mathalon, William S. Stone, Scott W. Woods, Elaine F. Walker, Diana O. Perkins, Hengyi Cao

**Affiliations:** a Department of Psychiatry and Behavioral Sciences, Emory University School of Medicine, 100 Woodruff Circle, Atlanta, GA 30322, USA; b Department of Psychiatry, Hotchkiss Brain Institute, University of Calgary, 3330 Hospital Drive NW, Calgary, AB T2N 4N1, Canada; c Departments of Psychiatry and Biobehavioral Sciences and Psychology, Semel Institute for Neuroscience and Human Behavior, University of California Los Angeles, 760 Westwood Plaza, Los Angeles, CA 90095, USA; d Department of Psychiatry, University of California San Diego School of Medicine, 9500 Gilman Drive, La Jolla, CA 92093, USA; e Department of Psychiatry, Yale University School of Medicine, 300 George Street, New Haven, CT 06511, USA; f Department of Psychology, Yale University, 100 College Street, New Haven, CT 06510, USA; g Division of Psychiatry Research, Zucker Hillside Hospital, 75-59 263^rd^ Street, Glen Oaks, NY 11004, USA; h Institute of Behavioral Science, Feinstein Institutes for Medical Research, 350 Community Drive, Manhasset, NY 11030, USA; i Department of Psychiatry, Donald and Barbara Zucker School of Medicine at Hofstra/Northwell, 500 Hofstra University, Hempstead, NY 11549, USA; j Department of Psychiatry, Harvard Medical School at Beth Israel Deaconess Medical Center, 330 Brookline Avenue, Boston, MA 02215, USA; k Department of Psychiatry & Behavioral Sciences, University of California, San Francisco, 675, 18th Street, San Francisco, CA 94143, USA; l San Francisco Veterans Affairs Health Care System, 4150 Clement Street, Building 203 Room, 1A-14, San Francisco, CA 94121, USA; m Department of Psychology, Emory University, 36 Eagle Row, Atlanta, GA 30322, USA; n Department of Psychiatry, University of North Carolina at Chapel Hill School of Medicine, 333, S Colombia Street, Suite 304, Chapel Hill, NC 27514, USA

**Keywords:** Adolescence, cerebello-thalamo-cortical connectivity (CTC), clinical high risk for psychosis (CHR-P), neurodevelopment, social fragmentation

## Abstract

Hyperconnectivity in the cerebello-thalamo-cortical (CTC) circuit, a key component of predictive coding, has been linked to schizophrenia and psychosis risk among youth at clinical high-risk for psychosis (CHR-P). The cerebellum monitors prediction errors, and its dysfunction may lead to misattributions of internal experiences and, consequently, hallucinations and delusions. Neighborhood social fragmentation, characterized by disrupted social ties and unpredictable norms, may compromise the brain’s ability to form stable models of the social world. Within predictive coding frameworks, such environments can hinder belief updating, increasing reliance on prior beliefs. This maladaptive process may disrupt CTC connectivity, thereby increasing vulnerability to psychosis. This study examined whether neighborhood social fragmentation predicted CTC connectivity in CHR-P and healthy comparison (HC) youth using functional connectivity data from the North American Prodrome Longitudinal Study Phase 2. Generalized linear mixed models assessed associations between social fragmentation and CTC connectivity, adjusting for age, sex, race/ethnicity, individual poverty, neighborhood educational attainment (proportion of residents with <9^th^ grade education), and study site. Participants (mean [SD] age = 19.46 [4.34]; 42.9 % female; 44.4 % White non-Hispanic) included 115 CHR-P and 74 HCs. CHR-P youth exhibited significantly greater CTC connectivity (mean [SD] = 0.28 [0.91]) compared to HC youth (mean [SD] = −0.44 [0.98]). Greater neighborhood social fragmentation was associated with increased CTC connectivity among CHR-P youth (adjusted β = 0.021, 95 % CI = 0.004–0.038), with a similar, nonsignificant trend among HCs (adjusted β = 0.016, 95 % CI = −0.009–0.041). These findings suggest that the social environment may contribute to psychosis risk by modulating CTC connectivity, highlighting potential targets for prevention and early intervention.

## Introduction

Growing evidence indicates that the cerebellum plays a central role in a range of higher-order cognitive functions beyond motor coordination. Through cerebello-thalamo-cortical (CTC) circuits linking the cerebellum and associative cortex via thalamic relays, the cerebellum is thought to generate and update internal models that support prediction and error monitoring, thereby facilitating coordinated higher-order processes such as sensory integration, directed attention, and cognitive control (Andreasen & Pierson, 2008; Badke D’Andrea et al., 2023; Middleton & Strick, 2001). Individuals with psychosis consistently demonstrate altered connectivity within this circuit (Forlim, Klock, Gallinat, & Kühn, 2024; Hengyi Cao et al., 2022; Wei et al., 2022; Yao et al., 2025), suggesting disruption of cerebellar predictive and error-monitoring mechanisms.

Notably, CTC network dysregulation, particularly hyperconnectivity, has also been observed in individuals at elevated risk for psychosis (Hengyi Cao et al., 2018; Hengyi Cao, Ingvar, Hultman, & Cannon, 2019; McGlashan, Walsh, & Woods, 2010), suggesting that aberrant CTC connectivity may precede the onset of psychosis and reflect early vulnerability-related alterations. This interpretation is consistent with the cognitive dysmetria theory (Andreasen, Paradiso, & O’Leary, 1998; Hengyi Cao & Cannon, 2019), which posits that disruptions to the cerebellum’s coordination of higher cognitive processes contribute to core impairments of schizophrenia. When the cerebellum’s role in prediction and error monitoring is compromised, internally generated experiences may be misattributed to external sources, giving rise to hallucinations and delusions (Andreasen & Pierson, 2008; Ide & Chiang-shan, 2011; Pinheiro, Schwartze, & Kotz, 2020; Yeganeh-Doost, Gruber, Falkai, & Schmitt, 2011).

Further, greater CTC connectivity has been linked to elevated risk for and subsequent transition to psychosis in individuals at clinical high-risk for psychosis (CHR-P) (Anticevic et al., 2015; Hengyi Cao et al., 2022). Youth at CHR-P, a risk state characterized by subthreshold psychotic symptoms or a combination of family history and functional decline (Addington et al., 2012; McGlashan et al., 2010), represent a critical population in which to examine these processes. Adolescence and emerging adulthood are periods of rapid brain maturation and heightened sensitivity to social context, coinciding with emergence of early psychotic experiences (Ordaz, Foran, Velanova, & Luna, 2013; Patel, Leathem, Currin, & Karlsgodt, 2021; Sanders et al., 2023; Spear, 2013; Uhlhaas et al., 2023). This developmental period thus offers a uniquely informative window for examining the neurobiological and contextual factors that shape vulnerability before illness onset, when preventative interventions may be most effective.

Neighborhood disadvantage is increasingly recognized as a critical social determinant of mental health, including risk for psychosis (Alegría, Alvarez, Cheng, & Falgas-Bague, 2023; Kirkbride, Jones, Ullrich, & Coid, 2014). Socioeconomic deprivation and social fragmentation are both common indicators of neighborhood disadvantage; however, they represent distinct constructs that capture different structural and social dimensions of place. While socioeconomic deprivation reflects material and economic disadvantage linked to social stratification, social fragmentation captures disruptions to community social organization and cohesion. Importantly, evidence indicates that these dimensions exert unique and largely independent effects on health and developmental outcomes (B. S. Ku, Pauselli, Manseau, & Compton, 2020; Martin et al., 2021). Consistent with this distinction, our prior work identified both shared and distinct pathways linking socioeconomic deprivation and social fragmentation to global social functioning, suggesting that these neighborhood characteristics may influence neurodevelopment through partially overlapping but also distinct mechanisms (Yuan et al., 2025).

Social fragmentation in particular has been consistently linked to psychosis incidence, symptom severity, and related structural brain alterations (Benson S. Ku et al., 2022; B. S. Ku, Addington, et al., 2021; B. S. Ku, Compton, Walker, & Druss, 2021; B. S. Ku et al., 2020; B. S. Ku, Walker, Druss, Murray, & Compton, 2023; Oluwoye, Puzia, Lissau, Amram, & Weeks, 2024; Solmi, Lewis, Zammit, & Kirkbride, 2020; Veling, Susser, Selten, & Hoek, 2015). Empirically, social fragmentation is most commonly operationalized as a composite index that aggregates multiple indicators of social disconnection, including residential instability and the proportions of single-person or single-parent households, unmarried adults, and renter-occupied housing (Aberizk et al., 2025; Allardyce et al., 2005; Benson S Ku et al., 2023; Congdon, 2024; Zammit et al., 2010). Together, these indicators reflect neighborhood-level conditions that may constrain stable social ties, collective efficacy, and social support.

Developmental exposure to socially fragmented neighborhoods may disrupt the brain’s ability to construct stable models of the social world. In such environments, individuals frequently encounter unfamiliar people and inconsistent social norms, reducing the predictability of sensory and social input. When external cues become too ambiguous to guide accurate interpretation, the brain may compensate by relying more heavily on prior beliefs to make sense of social experiences (Benson S Ku & Young, 2025). Within the framework of the predictive coding model of psychosis, these impairments to belief updating are thought to contribute to the development of psychotic symptoms and, ultimately, schizophrenia (Sterzer et al., 2018). Given the cerebellum’s role in predictive coding, social fragmentation may contribute to psychosis risk through maladaptive CTC connectivity.

The present study examined whether neighborhood social fragmentation was associated with CTC connectivity among adolescents at CHR-P and healthy comparison (HC) participants. We hypothesized that greater social fragmentation would be associated with greater CTC connectivity. We further hypothesized that this relationship would be stronger among the CHR-P group compared to HCs, reflecting CHR-P individuals’ heightened vulnerability to socioenvironmental stressors during adolescence (Benson S Ku et al., 2023). Ultimately, identifying risk factors that contribute to these aberrant connectivity patterns during this period may help clarify the mechanisms underlying psychosis risk and inform strategies for early intervention.

## Methods

### Participants

We derived baseline data from the North American Prodrome Longitudinal Study Phase 2 (NAPLS-2), a multisite consortium designed to investigate the early detection and prevention of psychosis in youth at CHR-P. The consortium included eight sites: Emory University, Harvard University, the University of Calgary, the University of California Los Angeles (UCLA), the University of California San Diego (UCSD), the University of North Carolina Chapel Hill (UNC), Yale University, and Zucker Hillside Hospital (ZHH). The analytic sample (*n* = 189) was recruited between 2009 and 2013 through referrals and community education initiatives. Detailed inclusion and exclusion criteria have been described previously (Addington et al., 2012). All participants provided written informed consent, and all study procedures were approved by the institutional review boards at each study site. For the current analyses, participants with missing data were excluded (eFig. 1).

### Sample characteristics

#### Sociodemographic characteristics

Sociodemographic characteristics, including age, sex, race/ethnicity, household income, household size, and current address, were self-reported at baseline (Addington et al., 2012). Individual poverty status was coded as a binary variable based on whether a participant’s household income fell below the 2014 U.S. Census poverty threshold adjusted for household size (LoPilato et al., 2021).

#### Neighborhood characteristics and social fragmentation

We geocoded participants’ self-reported addresses to obtain neighborhood (census tract)-level data. We derived neighborhood characteristics from the 2010 American Community Survey 5-year summary estimates (2006–2010)(Bureau, 2010). Target characteristics included the proportion of residents without at least a 9^th^ grade education, living alone, living in single-parent households, married, living in owner-occupied housing, and living in the same household as one year prior. Guided by the Congdon index of social fragmentation (Congdon, 1996) and related operationalizations, the latter five variables were standardized and averaged to derive a composite neighborhood social fragmentation index (Aberizk et al., 2025; Allardyce et al., 2005; Congdon, 2024; Benson S Ku et al., 2023). To ensure directional consistency across indicators, the latter three estimates were reverse-scored such that higher values reflected greater social fragmentation.

#### Multi-paradigm fMRI and connectivity derivation

CTC connectivity was derived from multi-paradigm fMRI data, as previously detailed, to account for heterogeneity in connectivity patterns across tasks (Hengyi Cao et al., 2018). Specifically, five fMRI paradigms were collected in the NAPLS-2 project: an eyes-open resting state paradigm, a verbal working memory task, a paired-associates memory encoding task, a paired-associates memory retrieval task, and an emotional face matching task. Data were acquired on either Siemens or General Electric 3T MR scanners using identical GRE-EPI protocols (TR/TE 2000/30 ms, flip angle = 77°, 30 slices, 4-mm thickness, 1-mm gap, 220 mm FOV). High-resolution T1-weighted images were obtained with MPRAGE (256 mm × 240 mm × 176 mm FOV, TR/TE 2300/2.91 ms, flip angle = 9°) or SPGR (260 mm FOV, TR/TE 7.0/minimum full ms, flip angle = 8°) sequences. Preprocessing followed standard SPM12 procedures.

As detailed in previous work demonstrating associations between CTC connectivity and psychosis-related outcomes (Hengyi Cao et al., 2018), cross-paradigm connectivity analysis was employed to calculate CTC connectivity (H. Cao et al., 2021). In brief, we extracted time series from each of the 270 regions defined by an expanded Power atlas (Power et al., 2011) during each paradigm. These time series were further corrected for motion, physiological signals, and mean effects of task-evoked coactivations (for task data), and were used in pairwise correlations to generate individual connectivity matrices. Paradigm-independent connectivity matrices were summarized using principal component analysis across all five paradigms. The previously identified CTC network was subsequently extracted from the connectivity matrices for each individual, which comprised a total of 84 links predominantly centered at the thalamus and posterior cerebellum. Further details regarding connectivity data acquisition and construction are described in prior literature (Hengyi Cao et al., 2018).

## Statistical analyses

### Descriptive statistics

We evaluated group differences between CHR-P and HC participants using independent samples *t*-tests for continuous variables and chi-square tests for categorical variables. We also employed these methods to compare descriptives between included and excluded participants (described in eFig. 1). Bivariate Pearson correlations examined unadjusted associations between covariates, social fragmentation, and CTC connectivity.

### Generalized linear mixed effects models

We assessed GLMM assumptions and identified no major violations; therefore, all predictors were retained in the final models (eTable 1). We used GLMMs to examine the associations between neighborhood social fragmentation and CTC connectivity. Univariable models first evaluated neighborhood social fragmentation as the sole predictor of CTC connectivity. Multivariable models then incorporated age, sex, White non-Hispanic race/ethnicity, individual poverty status, and neighborhood educational attainment as fixed effects covariates. Models were also estimated separately within the CHR-P and HC subgroups.

In addition, we fit a combined model including the full sample (*n* = 189) with an interaction term between neighborhood social fragmentation and CHR-P status to evaluate whether the association differed by clinical risk group.

### Sensitivity analyses

To assess whether observed associations were driven by specific components of the social fragmentation index, we conducted sensitivity analyses in which the primary model examining social fragmentation and CTC connectivity were re-estimated using each constituent indicator of social fragmentation separately (i.e., residential instability, single-person households, single-parent households, unmarried adults, and renter-occupied housing). These analyses were conducted separately within the CHR-P and HC groups.

Guided by the predictive coding framework underlying our hypothesis, we also conducted additional sensitivity analyses testing whether total positive or negative Scale of Prodromal Symptoms (SOPS) scores moderated the association between social fragmentation and CTC connectivity.

All models included a random intercept for study site, as attempts to include census tract resulted in nonconvergence. Robust covariance estimation was applied, and stringent convergence criteria were used to ensure model stability. All analyses were conducted using IBM SPSS Statistics (version 31.0) and RStudio (version 2025.09.2) (IBM Corp., 2023; Posit team, 2025; R Core Team, 2024).

## Results

### Participant characteristics

A total of 189 participants were included in the analytic sample (mean [SD] age = 19.46 [4.34]; 42.9 % female; 44.4 % White non-Hispanic), including 115 CHR-P youth and 74 HCs (eFig. 1, Table 1). Included and excluded participants did not differ meaningfully in demographic, neighborhood, or clinical characteristics (eTable 2). CHR-P and HC groups were comparable in age, sex distribution, race/ethnicity, and individual poverty status (Table 1). Effect sizes for group differences in neighborhood educational attainment and social fragmentation were small (|*d*| = 0.25–0.26), with no significant differences between groups.

CHR-P youth exhibited significantly greater CTC connectivity compared to HC youth (mean [SD] = 0.28 [0.91] for CHR-P; −0.44 [0.98] for HC; *t*(187) = 5.15, 95 % CI = 0.045 to 0.101, *d* = −0.77).

### Bivariate correlations

**Fig. 1.**
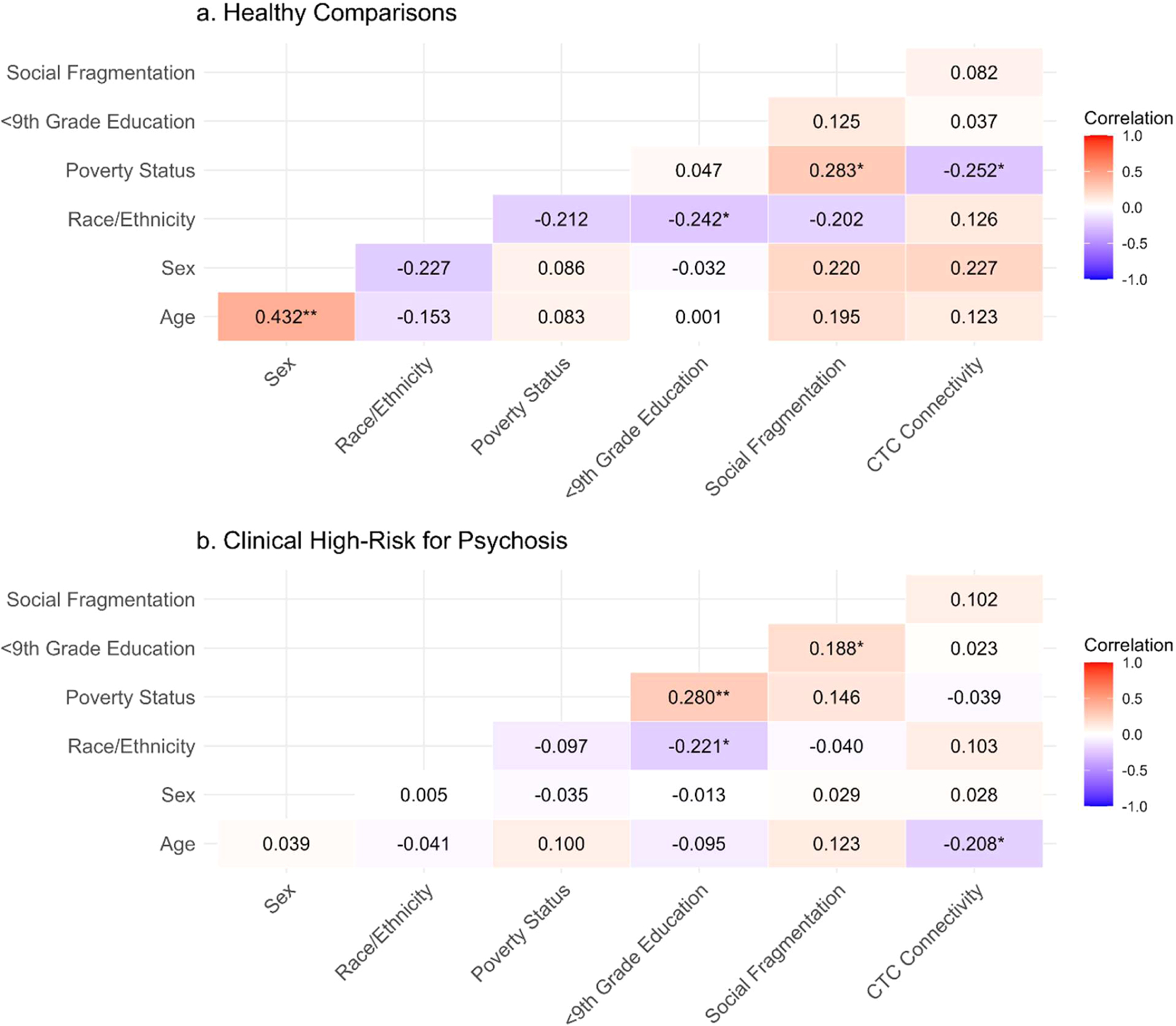
Within-group bivariate correlations among demographic, neighborhood, and cerebello-thalamo-cortical connectivity variables. *Abbreviations*. CHR-P, clinical high-risk for psychosis; CTC, cerebello-thalamo-cortical connectivity; HC, healthy control. *Notes*. Values represent Pearson correlation coefficients (*r*) between study variables. Neighborhood educational attainment reflects the proportion of residents within a census tract who completed less than a 9th grade education. Neighborhood social fragmentation is a composite measure including *z*-scored proportions of single-parent households, individuals living alone, owner-occupied housing (reverse scored), residential stability (reverse-scored), and married households (reverse scored) at the census tract level. CTC values represent standardized (z-scored) connectivity estimates. All significance tests are two-tailed. Significant *p*-values are indicated as *p* < .05 (*) and *p* < .01 (**).

Across both HC and CHR-P groups, White non-Hispanic race/ethnicity was associated with greater neighborhood educational attainment (*r* = −.242 and −.221, respectively; Fig. 1). Correlations were otherwise inconsistent across groups. No associations exceeded |*r*| = .500, further indicating acceptable linear independence among predictors for subsequent modeling. Bivariate correlations among study variables across the full analytic sample are presented in eFig. 2.

### Associations between neighborhood social fragmentation and CTC connectivity

**Fig. 2.**
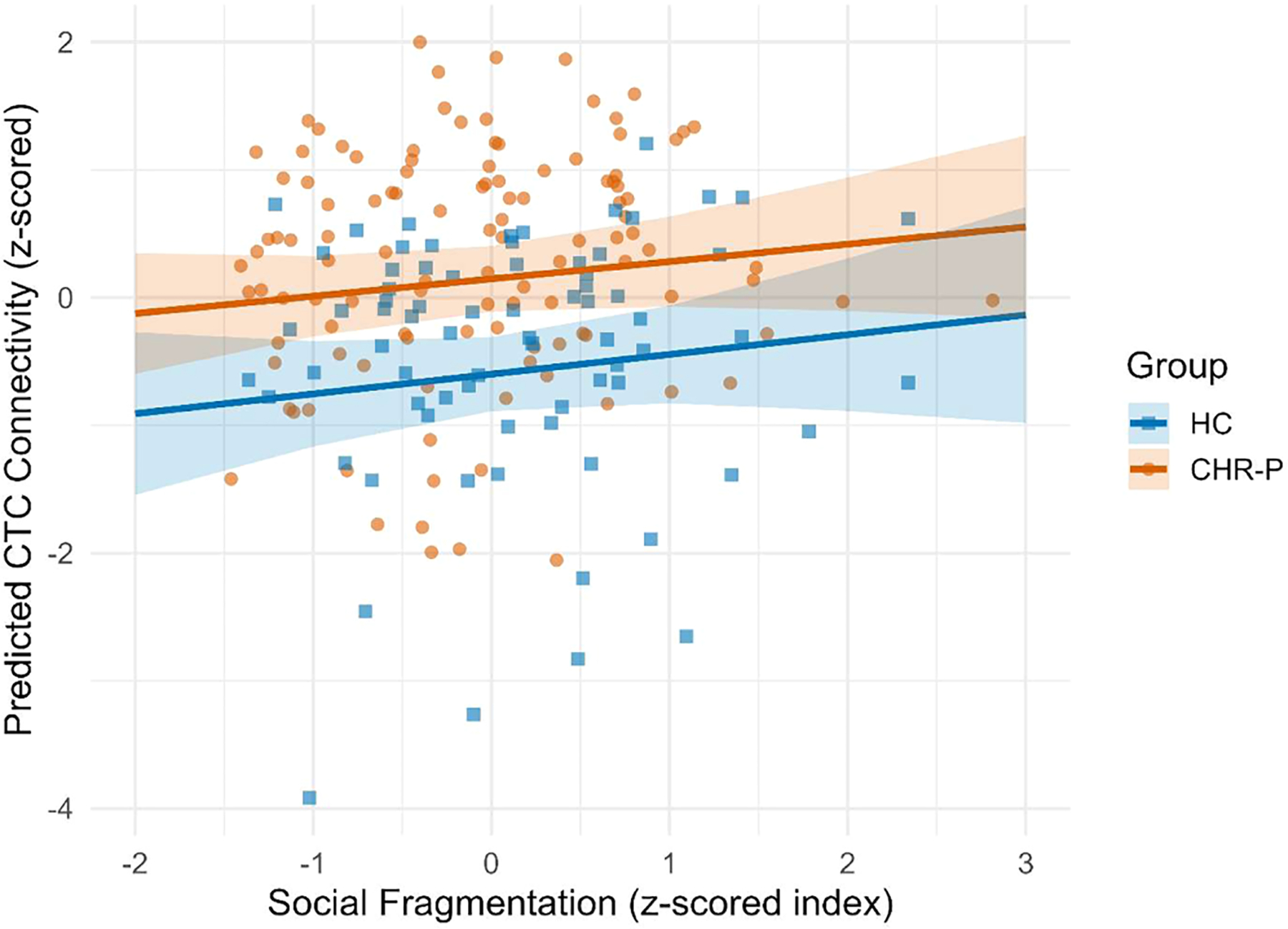
Association between social fragmentation and cerebello-thalamo-cortical connectivity by clinical high-risk status. *Abbreviations*. CHR-P, clinical high-risk for psychosis; CTC, cerebello-thalamo-cortical connectivity; HC, healthy control. *Notes*. The scatterplot illustrates the adjusted continuous association between neighborhood social fragmentation and cerebello-thalamo-cortical (CTC) connectivity separately for CHR-P (orange) and HC (blue) groups. Solid lines represent the fitted regression slopes with shaded 95 % confidence intervals, adjusted for age, sex, White non-Hispanic race/ethnicity, individual poverty, and neighborhood educational attainment (proportion of residents who obtained less than a 9th grade education). Neighborhood social fragmentation is a composite measure including *z*-scored proportions of single-parent households, individuals living alone, owner-occupied housing (reverse scored), residential stability (reverse-scored), and married households (reverse scored) at the census tract level. CTC values represent standardized (z-scored) connectivity estimates. Individual data points are overlaid. CHR-P youth had significantly greater CTC connectivity than HC (*t*(187) = 5.15, 95 % CI = 0.045 to 0.101).

**Fig. 3.**
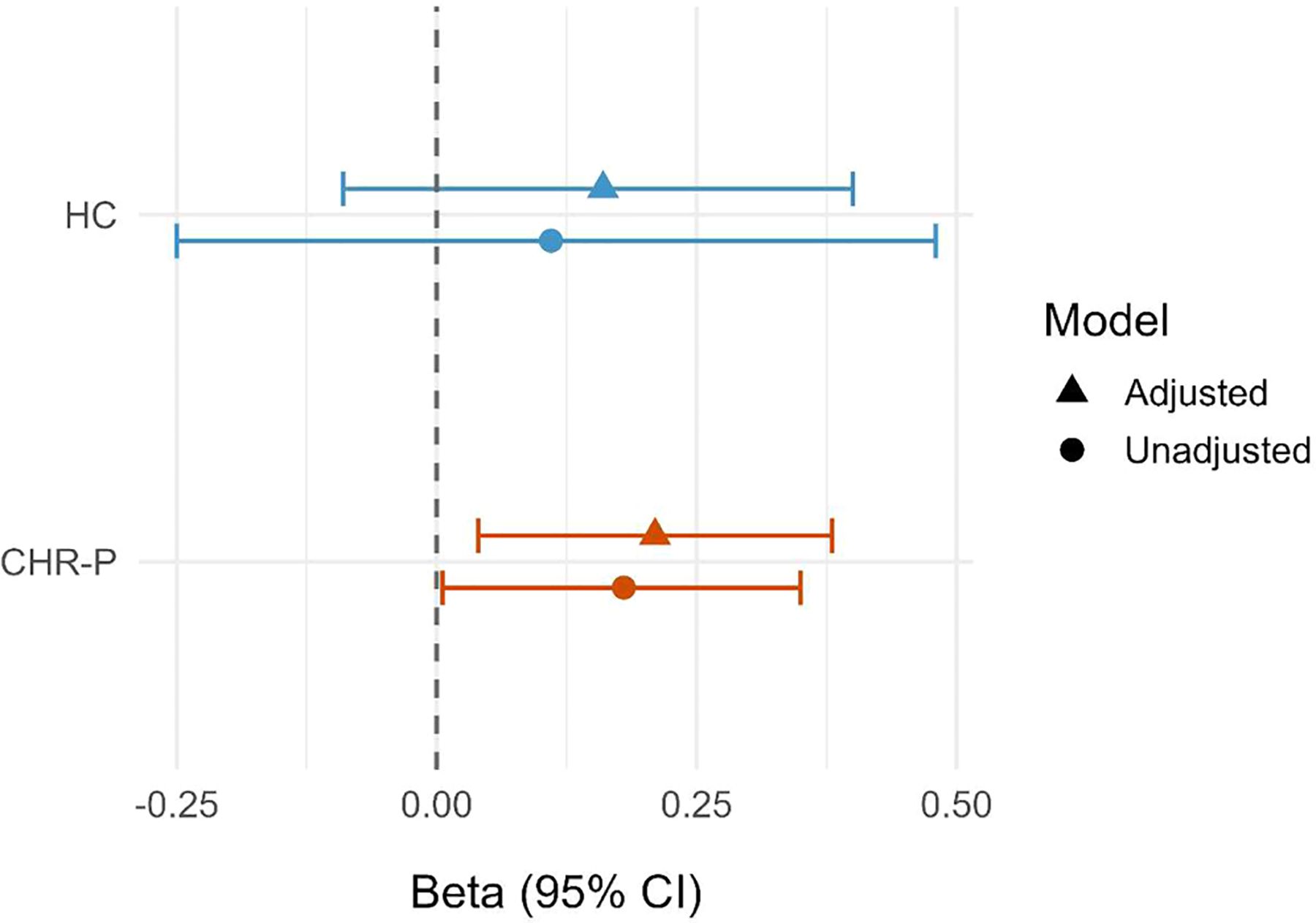
Adjusted and unadjusted associations between social fragmentation and cerebello-thalamo-cortical connectivity by clinical high-risk status. *Abbreviations*. CHR-P, clinical high-risk for psychosis; CI, confidence interval; HC, healthy control. *Notes*. The forest plot summarizes unadjusted and adjusted regression estimates for the association between neighborhood social fragmentation and cerebello-thalamo-cortical (CTC) connectivity, presented separately for the clinical high-risk (CHR) and healthy control (HC) groups. Points represent standardized regression coefficients (β), and horizontal lines indicate 95 % confidence intervals. Adjusted models controlled for age, sex, White non-Hispanic race/ethnicity, neighborhood-level education (% less than 9^th^ grade), and neighborhood poverty. CTC values represent standardized (z-scored) connectivity estimates. Corresponding regression coefficients, confidence intervals, and *p*-values are presented in eTable 3.

Scatterplots with group-specific regression lines revealed a positive linear relationship between neighborhood social fragmentation and CTC connectivity in both CHR-P and HC youth (Fig. 2). Among CHR-P participants, greater neighborhood social fragmentation was significantly associated with greater CTC connectivity in both unadjusted (β = 0.18, 95 % CI = 0.01 to 0.35) and adjusted models (β = 0.21, 95 % CI = 0.04 to 0.38; Fig. 3, eTable 3). This association was consistent, though not statistically significant, among HC youth (unadjusted β = 0.11, 95 % CI = −0.25 to 0.48; adjusted β = 0.16, 95 % CI = −0.09 to 0.40). Results were robust to alternative operationalizations of social fragmentation, with no indicators emerging as primary drivers of the association with CTC connectivity (eTable 4).

The interaction between CHR-P status and neighborhood social fragmentation was nonsignificant (adjusted β = −0.01, 95 % CI = −0.43 to 0.42; Table 2), indicating that the strength of the relationship between social fragmentation and CTC connectivity did not differ significantly between CHR-P and HC youth. The interaction between total positive and negative SOPS scores and neighborhood social fragmentation was similarly nonsignificant (eTable 5).

## Discussion

This study found that living in more socially fragmented neighborhoods during adolescence was associated with greater CTC connectivity, particularly among individuals at CHR-P. Prior work has independently linked both CTC hyperconnectivity and neighborhood social fragmentation to functional impairments and elevated psychosis risk (Benson S. Ku et al., 2025; B. S. Ku, Compton, et al., 2021; Hengyi Cao et al., 2018; Yao et al., 2025). Consistent with this literature, we observed greater CTC connectivity among CHR-P participants relative to HCs. Importantly, our study extends previous work by situating this neural pattern within the broader context of neighborhood social fragmentation. Together, our findings suggest that environmental stressors, such as social fragmentation, may contribute to neurodevelopmental vulnerability associated with psychosis risk.

Although the association between social fragmentation and CTC connectivity did not reach statistical significance among HCs, the comparable direction and magnitude of the effect suggest that this relationship may operate across groups. It is possible that the smaller HC sample (*n* = 74 vs. *n* = 115 for CHR-P) limited statistical power to detect effects of this magnitude (estimated power ≈ 0.55–.60 versus ≈ 0.80 for CHR-P). Further interaction analyses also indicated that the strength of the association between social fragmentation and CTC connectivity did not differ significantly by CHR-P status. This pattern is consistent with, but does not confirm, the possibility that social fragmentation relates to CTC connectivity in a similar manner across groups, with higher baseline CTC connectivity in CHR-P youth contributing to elevated risk (Hengyi Cao et al., 2022; Yao et al., 2025). However, modest group differences cannot be ruled out, particularly given the limited sample. Future research with larger samples will be needed to determine whether this positive relationship between social fragmentation and CTC connectivity is truly conserved or varied between risk groups.

Exposure to socially fragmented environments has been theorized to disrupt predictive coding processes by altering error signaling and belief updating (Benson S. Ku et al., 2025). At a molecular level, sustained social stress has been shown to influence γ-aminobutyric acid (GABA)-related gene expression, including reduced *GAD1* transcription and altered receptor signaling (Schmidt & Mirnics, 2015). Such perturbations in inhibitory tone may impair error detection and information integration, processes central to the “cognitive dysmetria” hypothesis of psychosis (Andreasen et al., 1998). Consistent with this framework, GABAergic interneuron dysfunction has been robustly implicated in psychotic disorders (Bullock, Bolognani, Botta, Valenzuela, & Perrone-Bizzozero, 2009; Yeganeh-Doost et al., 2011). Emerging evidence further implicates cerebellar GABA content as a mediator of CTC hyperconnectivity, directly linking inhibitory neurotransmission to the circuit-level dysregulation observed in psychosis (Yao et al., 2025). Together, these findings support a plausible pathway in which social stress-related alterations in GABAergic signaling contribute to impaired predictive processing and, in turn, aberrant CTC connectivity.

Although such adaptations may support stability within neural and social systems, they appear to do so at the expense of functional accuracy and, consequently, resilience to psychosis. Future research should continue to examine how biological and environmental factors interact across development to clarify their role in the pathogenesis of psychosis. Ultimately, these findings highlight both neural and environmental targets with potential translational significance for mitigating psychosis risk. For example, social cohesion and residential stability, particularly within at-risk populations, may represent modifiable upstream factors. Community-based interventions or investments in social infrastructure could help buffer against the adverse neurodevelopmental consequences of social fragmentation and, in turn, reduce vulnerability for psychosis.

### Limitations and future directions

Limitations should be considered when interpreting these findings. First, this study excluded participants due to missing data, potentially introducing bias; however, the excluded population did not differ from the analytic sample in sociodemographic characteristics. Second, the cross-sectional design precludes conclusions about causality or the temporal direction of effects between social fragmentation and CTC connectivity. Although our sample included youth at CHR-P with available transition data, the small number of individuals who transitioned to psychosis (*n* = 10) limited statistical power to examine associations among social fragmentation, CTC connectivity, and subsequent transition, precluding formal tests of the predictive validity. Larger, longitudinal studies will be critical to clarify the generalizability of this pattern beyond CHR-P populations and to confirm its persistence and utility across stages of illness progression.

Additionally, neighborhood-level social fragmentation was operationalized using participants’ baseline residential addresses, and information regarding residential duration was not available in NAPLS-2. As such, the present findings reflect neighborhood context at baseline rather than cumulative adolescent exposure to social fragmentation. These findings thus do not address whether timing or cumulative duration of exposure to socially fragmented environments across adolescence exerts differential or dose-dependent effects on CTC connectivity. Future longitudinal studies integrating residential history will be important for clarifying how the timing and duration of neighborhood exposure shape brain connectivity and psychosis risk. Such designs would also enable more granular investigation of the individual neighborhood characteristics that comprise social fragmentation, including whether specific indicators differentially contribute to risk and whether alternative or weighted indices more accurately capture the mechanisms linking neighborhood context to neurodevelopment.

In addition, recent evidence indicates that analyses distinguishing among specific cortical regions may provide valuable mechanistic insights into the CTC abnormalities observed in schizophrenia spectrum disorders (Lányi, 2025), highlighting the importance of additional regionally specific analytic approaches. Although the present study incorporated cerebellar nodes primarily located within the posterior cerebellum, providing some regional specificity, connectivity was ultimately summarized at the network level. Applying comparable cerebellar parcellation strategies may provide additional mechanistic insight into the heterogeneous cerebellar contributions to CTC dysconnectivity. Future studies should also explore how environmental factors influence dysconnectivity between these distinct brain regions.

## Conclusions

The present study demonstrates that greater social fragmentation is associated with increased CTC connectivity, suggesting a potential pathway through which early social adversity becomes biologically embedded and disrupts neurodevelopmental processes underlying cognitive and emotional coordination. By linking neighborhood-level social factors to neural function, this work highlights the importance of social determinants of health, with implications for developing effective targets for early intervention and prevention.

## Supplementary Material

supplementary materials

Supplementary material associated with this article can be found, in the online version, at doi:10.1016/j.braen.2026.100016.

## Figures and Tables

**Table 1:** Sociodemographic and neighborhood characteristics by clinical high-risk status.

	Overall	CHR-P	HC	p-value	Effect size
*n*	189	115	74		
Age (mean (SD))	19.46 (4.34)	19.33 (4.37)	19.66 (4.32)	.608	0.08
Female sex (*n* (%))	81 (42.9)	48 (41.7)	33 (44.6 %)	.813	0.03
White non-Hispanic (*n* (%))	84 (44.4)	50 (43.5)	34 (45.9 %)	.855	0.02
Below poverty line (*n* (%))	34 (18.0)	21 (18.3)	13 (17.6)	1.000	0.01
Neighborhood educational attainment (mean (SD))	6.23 (8.21)	7.03 (9.35)	4.98 (5.89)	.094	−0.25
Neighborhood social fragmentation (mean (SD))		−0.08 (0.82)	0.13 (0.81)	.081	0.26
Cerebello-thalamo-cortical connectivity (mean (SD))		0.28 (0.91)	−0.44 (0.98)	<0.001*	−0.77

*Abbreviations*. CHR-P, clinical high-risk for psychosis; HC, healthy control; SD, standard deviation.

*Notes*. Group comparisons between individuals at clinical high-risk (CHR) for psychosis and healthy controls (HC) were conducted using independent *t*-tests for continuous variables and chi-square tests for categorical variables. Neighborhood educational attainment reflects the proportion of residents within a census tract who completed less than a 9th grade education. Neighborhood social fragmentation is a composite measure including *z*-scored proportions of single-parent households, individuals living alone, owner-occupied housing (reverse scored), residential stability (reverse-scored), and married households (reverse scored) at the census tract level. CTC values represent standardized (z-scored) connectivity estimates. Effect sizes are reported as Cohen’s *d* for continuous variables and Cramér’s *V* for categorical variables.

**Table 2:** Univariable and multivariable linear mixed effects models of neighborhood social fragmentation on cerebello-thalamo-cortical connectivity including an interaction with clinical high-risk status.

Neighborhood social fragmentation	Unadjusted β	95 % CI	*p*-value
	0.12	0.06 to 0.19	<.001**
	Adjusted β	95 % CI	*p*-value
Age	−0.04	−0.02 to 0.02	.721
Sex	−0.23	−0.47 to 0.02	.067
Race/ethnicity	−0.21	−0.51 to 0.10	.189
Individual poverty	0.33	0.05 to 0.60	.019*
CHR-P status	−0.78	−0.92 to −0.64	<.001***
Neighborhood educational attainment	<0.01	−0.01 to 0.02	.602
Neighborhood social fragmentation	0.19	0.12 to 0.26	<.001***
	Interaction β	95 % CI	*p*-value
Age	>−0.01	−0.02 to 0.02	.721
Sex	−0.23	−0.50 to 0.04	.067
Race/ethnicity	−0.20	−0.50 to 0.09	.189
Individual poverty	0.33	0.04 to 0.62	.019*
CHR-P status	<0.01	−0.01 to 0.02	<.001***
Neighborhood educational attainment	−0.78	−0.92 to −0.64	.602
Neighborhood social fragmentation	0.19	<0.01 to 0.38	<.001***
Neighborhood social fragmentation*CHR-P status	−0.01	−0.43 to 0.42	.978

*Abbreviations*. CHR-P, clinical high-risk for psychosis; CI, confidence interval; CTC, cerebello-thalamo-cortical connectivity.

*Notes*. The univariable model examined the unadjusted association between neighborhood-level social fragmentation and CTC alone. Multivariable models adjusted for relevant covariates. Neighborhood educational attainment reflects the proportion of residents within a census tract who completed less than a 9th grade education. Neighborhood social fragmentation is a composite measure including *z*-scored proportions of single-parent households, individuals living alone, owner-occupied housing (reverse scored), residential stability (reverse-scored), and married households (reverse scored) at the census tract level. CTC values represent standardized (*z*-scored) connectivity estimates. An interaction term for social fragmentation by CHR-P status was included in the subsequent multivariable model. Significant *p*-values are indicated as *p* < .05 (*), *p* < .01 (**), and *p* < .001 (***).

## Data Availability

The data that support the findings of this study were obtained from the North American Prodrome Longitudinal Study Phase 2 (NAPLS-2) and access can be obtained upon request.
