# Supplementary material for: Neighborhood social fragmentation and cerebello-thalamo-cortical connectivity in youth at clinical high-risk for psychosis and healthy comparisons
Source: Brain Environ. Author manuscript; Available in PMC 2026 Mar 28. (PMC13030900; doi:10.1016/j.braen.2026.100016)
Supplement: supplementary materials [file NIHMS2158128-supplement-supplementary_materials.docx]

**Supplemental Materials**

Neighborhood social fragmentation and cerebello-thalamo-cortical connectivity in youth at clinical high-risk for psychosis and healthy comparisons.

Benson S. Ku, Ella J. Arrant, Jean Addington, Carrie E. Bearden, Kristin S. Cadenhead, Tyrone D. Cannon, Ricardo E. Carrion, Matcheri S. Keshavan, Daniel H. Mathalon, William S. Stone, Scott W. Woods, Elaine F. Walker, Diana O. Perkins, Hengyi Cao

**eTable 1.** Diagnostic tests for linear regression assumptions.

**eTable 2.** Demographic and neighborhood characteristics of included versus excluded participants.

**eTable 3.** Associations between neighborhood social fragmentation and cerebello-thalamo-cortical connectivity within clinical high-risk for psychosis and healthy comparison groups.

**eTable 4.** Sensitivity analyses examining associations between individual social fragmentation indicators and cortico-thalamo-cortical connectivity.

**eFigure 1.** Derivation of the analytic sample from North American Prodrome Longitudinal Study Phase 2 participants.

**eFigure 2.** Pearson correlations among demographic, neighborhood, and cerebello-thalamo-cortical variables within the full analytic sample.

**eTable 1.** Diagnostic tests for linear regression assumptions.

| Assumption | Test statistic | *df* | *p*-value | Interpretation |
| --- | --- | --- | --- | --- |
| Linearity | 0.76 | 180 | .450 | No evidence of nonlinearity |
| Homoscedasticity | 11.58 | 7 | .115 | No heteroscedasticity |
| Multicollinearity |  |  |  | All VIF < 2, no multicollinearity |
| Age | 1.08 |  |  |  |
| Sex | 1.05 |  |  |  |
| Race/ethnicity | 1.08 |  |  |  |
| Individual poverty | 1.09 |  |  |  |
| CHR-P status | 1.04 |  |  |  |
| Neighborhood education attainment | 1.13 |  |  |  |
| Neighborhood social fragmentation | 1.11 |  |  |  |

*Abbreviations.* CHR-P, clinical high-risk for psychosis; df, degrees of freedom; VIF, variance inflation factor.

*Notes.* Linear model assumption checks were conducted to evaluate the validity of the linear regression analyses. The Harvey-Collier test indicated no evidence of nonlinearity between the predictors (e.g., social fragmentation) and the outcome, cerebello-thalamo-cortical connectivity. The Breusch-Pagan test did not detect heteroscedasticity, supporting the assumption of equal error variance. VIF values for all variables were below 2, suggesting no meaningful multicollinearity. Taken together, thesis diagnostic checks indicate that the linear regression assumptions were adequately met for the reported models.

**eTable 2.** Demographic and neighborhood characteristics of included versus excluded participants.

|  | Overall | Included | Excluded | *p*-value | Effect size |
| --- | --- | --- | --- | --- | --- |
| *n* | 1895 | 189 | 1706 |  |  |
| Age (mean (SD)) | 18.84 (4.39) | 19.46 (4.34) | 18.70 (4.39) | .030* | −0.17 |
| Female sex (*n* (%)) | 466 (44.7) | 81 (42.9) | 385 (45.1) | .634 | 0.02 |
| White non-Hispanic (*n* (%)) | 524 (50.2) | 84 (44.4) | 440 (51.5) | .093 | 0.05 |
| Below poverty line (*n* (%)) | 187 (17.9) | 34 (18.0) | 153 (17.9) | 1.00 | <0.01 |
| CHR-P | 764 (73.3) | 115 (60.8) | 649 (76.0) | <.001*** | 0.13 |
| Neighborhood educational attainment (mean (SD)) | 5.89 (7.07) | 6.23 (8.21) | 5.76 (6.555) | .437 | −0.07 |
| Neighborhood social fragmentation (mean (SD)) |  | 0.08 (0.86) | −0.04 (0.80) | .076 | −0.15 |
| CTC connectivity (mean (SD)) |  | 0.01 (0.93) | −0.01 (1.04) | .866 | 0.02 |

*Abbreviations.* CHR-P, clinical high-risk for psychosis; CTC, cerebello-thalamo-cortical connectivity; SD, standard deviation.

*Notes.* Comparisons of demographic and sample characteristics between participants included in the analytic sample and those excluded due to missing data (see eFigure 1). Neighborhood educational attainment reflects the proportion of residents within a census tract who completed less than a 9^th^ grade education. Neighborhood social fragmentation is a composite measure including *z-*scored proportions of single-parent households, individuals living alone, owner-occupied housing (reverse scored), residential stability (reverse-scored), and married households (reverse scored) at the census tract level. CTC values represent standardized (*z-*scored) connectivity estimates. Significant *p*-values based on independent-samples *t*-tests for continuous variables and chi-square tests for categorical variables are indicated as *p*<.05 (*), *p*<.01 (**), and *p*<.001 (***). Effect sizes are reported as Cohen’s *d* for continuous variables and Cramér’s *v* for categorical variables.

**eTable 3.** Associations between neighborhood social fragmentation and cerebello-thalamo-cortical connectivity within clinical high-risk for psychosis and healthy comparison groups.

|  | CHR-P (*n*=115) | | | HC (*n*=74) | | |
| --- | --- | --- | --- | --- | --- | --- |
|  | Unadjusted β | 95% CI | *p*-value | Unadjusted β | 95% CI | *p*-value |
| Neighborhood social fragmentation | 0.18 | 0.01 to 0.35 | 0.044 | 0.11 | −0.25 to 0.48 | .540 |
|  | Adjusted β | 95% CI | *p*-value | Adjusted β | 95% CI | *p*-value |
| Age | −0.03 | −0.05 to <0.00 | .032* | 0.01 | −0.02 to 0.04 | .514 |
| Sex | −0.06 | −0.36 to 0.23 | .670 | −0.47 | −1.06 to 0.12 | .114 |
| Race/ethnicity | −0.16 | −0.52 to 0.22 | .408 | −0.33 | −0.82 to 0.15 | .174 |
| Individual poverty | 0.09 | −0.24 to 0.42 | .591 | 0.70 | 0.35 to 1.04 | <.001*** |
| Neighborhood educational attainment | −0.01 | −0.01 to 0.003 | .204 | 0.02 | −0.03 to 0.06 | .474 |
| Neighborhood social fragmentation | 0.21 | 0.04 to 0.38 | .018* | 0.16 | −0.09 to 0.40 | .218 |

Abbreviations: CHR-P, clinical high-risk for psychosis; CI, confidence interval; HC, healthy control.

Notes: Results from generalized linear mixed models examining the association between social fragmentation and cerebello-thalamo-cortical connectivity (CTC) separately within CHR-P and HC groups. The univariable model examined the unadjusted association between neighborhood social fragmentation and CTC connectivity. Adjusted models included age, sex, White non-Hispanic race/ethnicity, neighborhood level-education attainment (less than 9^th^ grade), and individual poverty as fixed covariates. Neighborhood educational attainment reflects the proportion of residents within a census tract who completed less than a 9^th^ grade education. Neighborhood social fragmentation is a composite measure including *z-*scored proportions of single-parent households, individuals living alone, owner-occupied housing (reverse scored), residential stability (reverse-scored), and married households (reverse scored) at the census tract level. CTC values represent standardized (*z-*scored) connectivity estimates. All models included study site as a random intercept. Significance levels are indicated as follows: Significant *p*-values are indicated as *p*<.05 (*), *p*<.01 (**), and *p*<.001 (***).

**eTable 4.** Sensitivity analyses examining associations between individual social fragmentation indicators and cortico-thalamo-cortical connectivity.

|  | CHR-P (*n*=115) | | | HC (*n*=74) | | |
| --- | --- | --- | --- | --- | --- | --- |
|  | β | 95% CI | *p*-value | β | 95% CI | *p*-value |
| **Neighborhood social fragmentation** | 0.21 | 0.04 to 0.38 | .018* | 0.16 | −0.09 to 0.40 | .218 |
| Living alone | 0.14 | 0.05 to 0.24 | .003** | 0.10 | −0.10 to 0.29 | .328 |
| Single-parent households | 0.19 | 0.05 to 0.34 | .010* | 0.10 | −0.11 to 0.31 | .350 |
| Unmarried | 0.19 | 0.08 to 0.30 | <.001*** | 0.11 | −0.15 to 0.37 | .395 |
| Residential instability | 0.03 | −0.06 to 0.12 | .552 | −0.05 | −0.21 to 0.12 | .594 |
| Renter housing | 0.19 | 0.04 to 0.35 | .017* | 0.27 | 0.15 to 0.39 | <.001*** |

*Abbreviations:* CHR-P, clinical high-risk for psychosis; CI, confidence interval; HC, healthy comparisons.

*Notes.* For ease of comparison, the association between the composite social fragmentation index and CTC connectivity from the main model is reproduced in the first row of the table. Subsequent rows report results from sensitivity analyses examining each constituent indicator separately. Models included age, sex, White non-Hispanic race/ethnicity, neighborhood level-education attainment (less than 9^th^ grade), and individual poverty as fixed covariates (covariate estimates not reported in the table). Significant *p*-values are indicated as *p*<.05 (*), *p*<.01 (**), and *p*<.001 (***)*.*

**eTable 5.** Sensitivity analysis examining positive and negative psychosis-risk symptom severity as a moderator of neighborhood social fragmentation—cerebello-thalamo-cortical connectivity associations.

|  | Interaction β | 95% CI | *p*-value |
| --- | --- | --- | --- |
| Age | -0.01 | -0.02 to 0.01 | .429 |
| Sex | -0.22 | -0.53 to 0.09 | .169 |
| Race/ethnicity | -0.21 | -0.51 to 0.09 | .167 |
| Individual poverty | 0.29 | 0.01 to 0.56 | .044* |
| Total positive SOPS | >0.00 | -0.01 to 0.01 | .685 |
| Neighborhood educational attainment | 0.29 | 0.19 to 0.38 | <.001*** |
| Neighborhood social fragmentation | 0.19 | 0.13 to 0.25 | <.001*** |
| Neighborhood social fragmentation × Total positive SOPS | -0.03 | -0.18 to 0.12 | .713 |
| Age | <0.00 | -0.02 to 0.01 | .692 |
| Sex | -0.22 | -0.50 to 0.06 | .122 |
| Race/ethnicity | -0.20 | -0.51 to 0.12 | .217 |
| Individual poverty | 0.34 | 0.04 to 0.63 | .025* |
| Total negative SOPS | 0.01 | -0.01 to 0.02 | .372 |
| Neighborhood educational attainment | 0.27 | 0.19 to 0.34 | <.001*** |
| Neighborhood social fragmentation | 0.17 | 0.09 to 0.25 | <.001*** |
| Neighborhood social fragmentation × Total negative SOPS | -0.05 | -0.13 to 0.02 | .157 |

*Abbreviations.* CI, confidence interval; SOPS, Scale of Prodromal Symptoms.

*Notes.* β values represent fixed-effect estimates from mixed-effects models including a random intercept for site. Symptom severity was indexed using the Scale of Prodromal Symptoms; positive and negative symptom domains were examined in separate models. Continuous predictors were *z*-scored prior to inclusion. All models adjusted for age, sex, race/ethnicity, individual-level poverty, and neighborhood-level educational attainment. Significant *p*-values are indicated as *p*<.05 (*), *p*<.01 (**), and *p<*.001 (***)*.*

**eFigure 1.** Derivation of the analytic sample from North American Prodrome Longitudinal Study Phase 2 participants.

Participants without CTC connectivity data (*n* = 462)

NAPLS 2 Participants

*N = 1043*

*n* = 651

**Final included sample**

***N* = 189**

**CHR-P**

***n* = 115**

**HC**

***n* = 74**

Participants without available neighborhood-level data (*n* = 392)

*Abbreviations.* CHR-P, clinical high-risk for psychosis; CTC, cerebello-thalamo-cortical connectivity; HC, healthy control; NAPLS-2, North American Prodrome Longitudinal Study 2.

*Notes.* The flow chart illustrates the derivation of the analytic sample. Neighborhood-level data were census tract-level proportions, including educational attainment (less than 9^th^ grade), single-parent households, living alone, residential stability, and owner-occupied housing. For a given participant, neighborhood characteristics were either all available or all missing due to the geocoding process.

**eFigure 2.** Pearson correlations among demographic, neighborhood, and cerebello-thalamo-cortical connectivity variables within the full analytic sample.


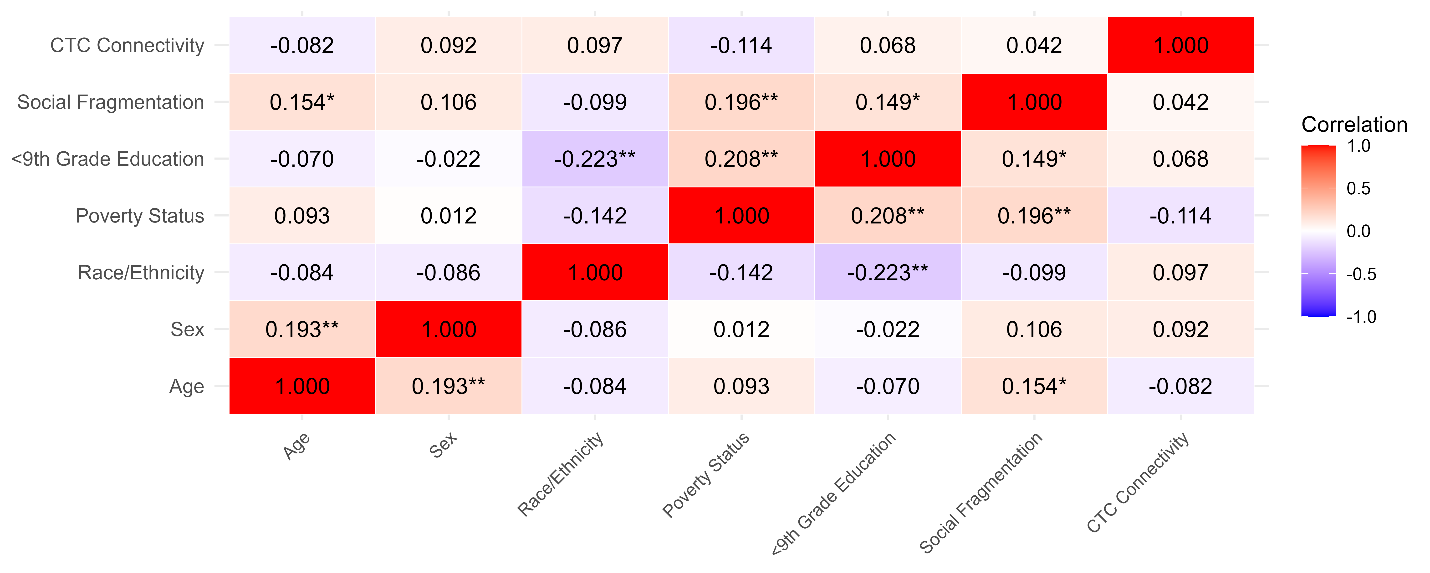


*Abbreviation.* CTC, cerebello-thalamo-cortical connectivity.

*Notes.* Values represent Pearson correlation coefficients (*r*) between study variables. Neighborhood educational attainment reflects the proportion of residents within a census tract who completed less than a 9^th^ grade education. Neighborhood social fragmentation is a composite measure including *z-*scored proportions of single-parent households, individuals living alone, owner-occupied housing (reverse scored), residential stability (reverse-scored), and married households (reverse scored) at the census tract level. CTC values represent standardized (*z-*scored) connectivity estimates. All significance tests are two-tailed. Significance levels are indicated as follows: **p*<.05, ***p*<.01.
